# Supplementary material for: Reproductive tract and pouch anatomical variability across the reproductive phases in female common opossum (Didelphis marsupialis Linnaeus, 1758)
Source: PLoS One. 2025 Oct 9;20(10):e0334040. doi: 10.1371/journal.pone.0334040 (PMC12510574; doi:10.1371/journal.pone.0334040)
Supplement: Attachment — Submitted filename: PONE-D-25-30424_Review.docx [file pone.0334040.s001.docx]

Manuscript PONE-D-25-30424

**Reproductive Tract and Pouch Anatomical Variability Across the Reproductive Phases in female common opossum (*Didelphis marsupialis* Linnaeus, 1758)**

The authors present a comprehensive, coherent, and well-written study on the female reproductive system of *Didelphis marsupialis*. A considerable number of female specimens at different stages of the reproductive cycle were used to carry out this study; however, the number of pregnant females was limited, and no individuals in estrus were included. This may be considered its most significant limitation.

This is a well-documented study, featuring high-quality figures and diagrams, and supported by appropriate references in both quantity and relevance.

Apart from the point already mentioned, no major flaws are observed in the study’s design, development, results, discussion, or conclusions. Therefore, the comments provided below may be considered minor and are intended to help improve the manuscript.

**L1:** Short title: Reproductive variability in common opossum. To make the short title more informative, it should include a reference to the fact that it concerns the female reproductive system, such as: ‘Female reproductive variability in common opossum’, for example.

**L21:** In the abstract, the first time the species name appears, it must be written in full (without abbreviation).

L58-59: To avoid repeating ‘among’ in the same sentence, it can be rewritten as: …’numerous anatomical variations occur at both the interspecific and intraspecific levels.’

**L73:** Avoid repeating ‘improve’ in the same sentence.

**L79-80:** The sentence could be improved for clarity: ‘Thus, this knowledge could enhance their reproductive strategies, including the use of biotechnologies’ or a comparable alternative.

**L109:** The name for ‘the back of the dorsal part of the neck’= ‘nape’ may be replaced with a more anatomical term that conveys a more scientific tone, such as ‘*nucha*’. This term could be changed in table 1 as well: ‘*nucha*-cloaca’ instead of ‘nape-cloaca’.

**L110-111:** The sentence is redundant because it states twice the absence of canine teeth.

**L112:** Delete ‘a’ from: …the presence of ‘**a**’ canines…

**L113-114:** The dental formula should include the initials/abbreviarions of each type of tooth.

**L147:** The plural of *corpus luteum* is *corpora lutea*.

**L175:** There are very few subadult and juvenile individuals; therefore, the data in Table 1 referring to these age groups should be interpreted with caution.

**L198-199:** Figure 2 legend: the urinary bladder is labeled as ‘**Ub**’, not as ‘**B**’. The same applies to Figures 6 and 8.

**L203:** Figure 2 legend: the label '**Ur**' does not appear in the images, and the element indicated by one of the **white arrows** in panel D is not specified.

**L207-208:** “**Caudally**, the ligament fanned out with fibers extending towards the mesocolon, kidney, and ureter.” Is the orientation of the suspensory ligament of the ovary accurately described, or would '**cranially**' be more appropriate, considering that its caudal extremity is the one anchored to the ovary?

**L218:** “(B) Microphotograph of the uterine tube.” To make it more informative, the phrase 'displaying its epithelial lining' can be added to the legend.

**L221:** …”most of them had ovaries with no visible structures (n=36, 71.9%), while the other females exhibited”…

To be more accurate, it could say: ‘most of them had ovaries with, **apparently,** no visible structures **(follicles/ corpora lutea)** (n=36, 71.9%), while the other females exhibited”…

**L227:** …’revealed follicles at all stages’…. To be more accurate, it should say ’revealed follicles at all stages **of development**,’…

**L234:** Figure 4 legend: corpus luteum in plural= ***corpora lutea***.

**L240:** Figure 5 legend: What is indicated by the **black arrow** in image (A), which lacks a corresponding label in the legend?

Table 2: On the left column, it is recommended that the term 'pregnancy' be indented beneath 'diestrus', since pregnancy constitutes a phase within diestrus.

L262: Figure 6 legend: as noted for Figure 2, the urinary bladder is labeled as '**Ub**', not '**B**'.

L264: Figure 6 legend: Instead of 'Fclv, folds of the cranial lateral vagina', it would be more accurate to state 'Fclv, **mucosal** folds of the cranial lateral vagina'.

**L265:** Figure 6 legend: instead of ‘Iul, interuterino ligament’, it should say ‘Iul, interuterin**e** ligament’.

**L282-284:** The term 'individuals' appears four times over the span of three lines. It is recommended to use appropriate synonyms to enhance clarity and avoid redundancy.

**L282-283:** …’while the remaining 38 individuals exhibited a vaginal sinus without divisions, with the presence of **a cranial fold** in all the individuals?...

With reference to this cranial mucosal fold, observed in 38 of the 57 specimens, is it situated **dorsally** or **ventrally**? Based on Figure 7, diagrams 2–6, the fold of the vaginal sinus (**Vsf**) depicted in **cross-section A** is positioned opposite the urethra (**Ur**) shown in **cross-section C**, which implies a dorsal location. It would be advisable to describe its precise position in this paragraph, given that in other species it is situated ventrally.

**L286-287:** The term 'individuals' is repeated once again: …“all **individuals** with a septum had a double and short cul-de-sac, whereas the other **individuals** could present variations in the cul-de-sac morphology”…

With regard to the underlined text, and in order to clarify the information further, it could be rewritten as follows:… whereas the other **specimens lacking a septum** could present variations…

**L298:** Figure 7 legend:… “**U**, uterus; **U**, urethra”… The urethra is labeled as '**Ur**' in the transverse sections C.

Regarding **Figure 7 quality**, the image would be significantly improved if the diagrams had greater contrast.

**L325 :** … ‘the ascending vagina, which was thin, oriented cranially, and included both a fused section attached to the urogenital cord and a free portion; and the descending vagina, which was thicker and ended at the vaginal sinus.

To make the text more informative, it may be rewritten as follows:…the ascending vagina, which was thin, oriented cranially, and included both a fused section attached to the urogenital cord and a free portion **oriented craneolaterally**, **followed by** the descending vagina, which was thicker and ended at the vaginal sinus.

**L337:** Figure 8 legend: ‘**Ub**’ for bladder, not ‘B’.

**L337:** Figure 8 legend: To complement the explanation of **image B**, the following or a similar statement may be added: “To visualize the lumen of the lateral vaginas, two rounded metal probes were inserted in a caudal-to-cranial direction, while a flattened probe was introduced in the same orientation into the centrally positioned birth canal.”

**L346:** Figure 9 legend: Before explaining the labels, it may be added: “As an anatomical reference, the urethra runs ventrally” or a similar statement.

**L361-362:** ‘E. Detail of the regressing birth canal’. It may be completed adding: E. Detail of the regressing birth canal **displaying its mucosa at higher magnification.**

**L368:** …’it had a bifid glans and a groove extending from the bifurcation to the middle portion of the glans’. For further clarification, the following can be added: ’it had a bifid glans **oriented caudolaterally,** and’…

**L372-373:** ‘B. Detail of the clitoris. B, Base of the glans;… To avoid confusion with the image label, it could be designated as 'Ba' instead of 'B'. Additionally, to provide more information, the following may be added: Base of the glans ‘**oriented cranially’**…

**L376:** Instead of ‘Ugcf, folds of the urogenital canal’, it should read 'Ugcf, **mucosal** folds of the urogenital canal'

**L385:** … ‘In one juvenile and two adults, no teats could be identified, and smooth skin was found inside the skin fold (Figure 12C).’ The morphological description is shown in image **12D**, not in image 12C.

**L395-397:** ‘Regarding the number and arrangement of teats, significant variability was found, ranging from 0 to 11 teats, which could be button-shaped, papillary-shaped, or fully elongated.’ To complement the information provided, the following may be added: …**’depending on their physiological state**’: ‘Regarding the number and arrangement of teats, significant variability was found, ranging from 0 to 11 teats, which could be button-shaped, papillary-shaped, or fully elongated **depending on their physiological state’**…

**L406**: …‘while both subadults and juveniles had **no teats**.’… It should be clarified whether it is ‘**no teats’** or ‘**no inactive teats’.**

**L410:** …’In contrast, adult females had a considerably lower mean of 1.29 ± 2.26’. It should be added: In contrast, adult females had a considerably lower mean of **undelevolped teats** **(**1.29 ± 2.26**).**

**L412**: Table 3 is not included in the manuscript

**L417:** … observing that the reproductive tract of *Didelphis marsupialis*… It may be appropriate to include the term 'female': …observing that the **female** reproductive tract of *Didelphis marsupialis…*

**L468:** It would be advisable to begin the sentence with: **'Regarding the oviduct’**, marsupials are reported to have…

**L495:** ‘In *D. marsupialis*, the perforated septum formed a vaginal sinus fold in the cranial portion’,… This vaginal sinus fold is placed dorsal and ventral, only dorsal or only ventral? Please, provide further specification or clarification.

**L530:** … ‘These findings suggest that the presence of epithelium in the birth canal may represent an intraspecific variation among marsupials.’ Only intraspecific? Or Intraspecific & interspecific?

Regarding the **References** (from **L610 onwards**):

Please review the hyphens used in the pagination, as some are short and others are long, which may indicate inconsistent usage. The same issue applies to the journal names: while most are written in full, some appear in abbreviated form (#5, 16).

Reference #2: Remove the parentheses from the pagination.

Reference #14: Incomplete. Is it a book chapter or a journal article?

Reference #24: Add parentheses around the year of publication.
