# Supplementary material for: Reproductive tract and pouch anatomical variability across the reproductive phases in female common opossum (Didelphis marsupialis Linnaeus, 1758)
Source: PLoS One. 2025 Oct 9;20(10):e0334040. doi: 10.1371/journal.pone.0334040 (PMC12510574; doi:10.1371/journal.pone.0334040)
Supplement: Attachment — Submitted filename: Response to reviewers.docx [file pone.0334040.s002.docx]

Manuscript PONE-D-25-30424

**Reviewer #1**

The corrections in the manuscript were highlighted in yellow.

**Reproductive Tract and Pouch Anatomical Variability Across the Reproductive Phases in female common opossum (*Didelphis marsupialis* Linnaeus, 1758)**

The authors present a comprehensive, coherent, and well-written study on the female reproductive system of *Didelphis marsupialis*. A considerable number of female specimens at different stages of the reproductive cycle were used to carry out this study; however, the number of pregnant females was limited, and no individuals in estrus were included. This may be considered its most significant limitation.

This is a well-documented study, featuring high-quality figures and diagrams, and supported by appropriate references in both quantity and relevance.

Apart from the point already mentioned, no major flaws are observed in the study’s design, development, results, discussion, or conclusions. Therefore, the comments provided below may be considered minor and are intended to help improve the manuscript.

**L1:** Short title: Reproductive variability in common opossum. To make the short title more informative, it should include a reference to the fact that it concerns the female reproductive system, such as: ‘Female reproductive variability in common opossum’, for example.

Done.

**L21:** In the abstract, the first time the species name appears, it must be written in full (without abbreviation).

Done.

L58-59: To avoid repeating ‘among’ in the same sentence, it can be rewritten as: …’numerous anatomical variations occur at both the interspecific and intraspecific levels.’

Done.

**L73:** Avoid repeating ‘improve’ in the same sentence.

Done.

**L79-80:** The sentence could be improved for clarity: ‘Thus, this knowledge could enhance their reproductive strategies, including the use of biotechnologies’ or a comparable alternative.

Done.

**L109:** The name for ‘the back of the dorsal part of the neck’= ‘nape’ may be replaced with a more anatomical term that conveys a more scientific tone, such as ‘*nucha*’. This term could be changed in table 1 as well: ‘*nucha*-cloaca’ instead of ‘nape-cloaca’.

Done.

**L110-111:** The sentence is redundant because it states twice the absence of canine teeth.

Done.

**L112:** Delete ‘a’ from: …the presence of ‘**a**’ canines…

Done.

**L113-114:** The dental formula should include the initials/abbreviarions of each type of tooth.

Done.

**L147:** The plural of *corpus luteum* is *corpora lutea*.

Done.

**L175:** There are very few subadult and juvenile individuals; therefore, the data in Table 1 referring to these age groups should be interpreted with caution.

We thank the reviewer for this important observation. As noted, the number of subadult and juvenile individuals in the sample is limited, and we agree that the data referring to these age groups in Table 1 should be interpreted with caution. The specimens used in this study were obtained through donations from regional environmental agencies, and availability depends entirely on the animals received by these institutions. In this case, we received a larger number of adult specimens and only a few subadults and juveniles. Nevertheless, we chose to include these younger individuals in the analysis to take advantage of the opportunity to examine their reproductive anatomy, while remaining fully aware of the limitations imposed by the small sample size.

**L198-199:** Figure 2 legend: the urinary bladder is labeled as ‘**Ub**’, not as ‘**B**’. The same applies to Figures 6 and 8.

Done.

**L203:** Figure 2 legend: the label '**Ur**' does not appear in the images, and the element indicated by one of the **white arrows** in panel D is not specified.

Done.

**L207-208:** “**Caudally**, the ligament fanned out with fibers extending towards the mesocolon, kidney, and ureter.” Is the orientation of the suspensory ligament of the ovary accurately described, or would '**cranially**' be more appropriate, considering that its caudal extremity is the one anchored to the ovary?

The description refers to the caudal portion of the suspensory ligament of the ovary, which fans out toward the mesocolon, kidney, and ureter. As observed in our dissections, the suspensory ligament in *D. marsupialis* can be divided into two portions: a cranial portion associated with the cranial part of the ureter, and a caudal portion that spreads out in a fan-like manner toward the surrounding structures. We have reviewed the wording to ensure clarity and anatomical accuracy in the description.

**L218:** “(B) Microphotograph of the uterine tube.” To make it more informative, the phrase 'displaying its epithelial lining' can be added to the legend.

Done.

**L221:** …”most of them had ovaries with no visible structures (n=36, 71.9%), while the other females exhibited”…

Done.

To be more accurate, it could say: ‘most of them had ovaries with, **apparently,** no visible structures **(follicles/ corpora lutea)** (n=36, 71.9%), while the other females exhibited”…

Done.

**L227:** …’revealed follicles at all stages’…. To be more accurate, it should say ’revealed follicles at all stages **of development**,’…

Done.

**L234:** Figure 4 legend: corpus luteum in plural= ***corpora lutea***.

Done.

**L240:** Figure 5 legend: What is indicated by the **black arrow** in image (A), which lacks a corresponding label in the legend? Se borró la flecha, porque no faltaba nada por señalar.

Done.

Table 2: On the left column, it is recommended that the term 'pregnancy' be indented beneath 'diestrus', since pregnancy constitutes a phase within diestrus.

We appreciate the reviewer’s suggestion. We agree that pregnancy is a phase within diestrus, and this is clearly stated in the text. However, the only pregnant female in our study was always considered separately in the analyses. Indenting "pregnancy" under "diestrus" in Table 2 could imply that the pregnant individual is included within the seven diestrus females, which is not the case. Therefore, we chose to keep pregnancy as a separate category to avoid potential misunderstanding.

L262: Figure 6 legend: as noted for Figure 2, the urinary bladder is labeled as '**Ub**', not '**B**'.

Done.

L264: Figure 6 legend: Instead of 'Fclv, folds of the cranial lateral vagina', it would be more accurate to state 'Fclv, **mucosal** folds of the cranial lateral vagina'.

Done.

**L265:** Figure 6 legend: instead of ‘Iul, interuterino ligament’, it should say ‘Iul, interuterin**e** ligament’.

Done.

**L282-284:** The term 'individuals' appears four times over the span of three lines. It is recommended to use appropriate synonyms to enhance clarity and avoid redundancy.

Done.

**L282-283:** …’while the remaining 38 individuals exhibited a vaginal sinus without divisions, with the presence of **a cranial fold** in all the individuals?...

With reference to this cranial mucosal fold, observed in 38 of the 57 specimens, is it situated **dorsally** or **ventrally**? Based on Figure 7, diagrams 2–6, the fold of the vaginal sinus (**Vsf**) depicted in **cross-section A** is positioned opposite the urethra (**Ur**) shown in **cross-section C**, which implies a dorsal location. It would be advisable to describe its precise position in this paragraph, given that in other species it is situated ventrally.

Done.

**L286-287:** The term 'individuals' is repeated once again: …“all **individuals** with a septum had a double and short cul-de-sac, whereas the other **individuals** could present variations in the cul-de-sac morphology”…

With regard to the underlined text, and in order to clarify the information further, it could be rewritten as follows:… whereas the other **specimens lacking a septum** could present variations…

Done.

**L298:** Figure 7 legend:… “**U**, uterus; **U**, urethra”… The urethra is labeled as '**Ur**' in the transverse sections C.

Done.

Regarding **Figure 7 quality**, the image would be significantly improved if the diagrams had greater contrast.

Thank you for your comment. While we agree that increasing the contrast could improve clarity, due to limitations of the original image/source data, further adjustments are not possible without risking loss of accuracy. We have optimized the figure to the fullest extent feasible and hope it is acceptable in its current form.

**L325 :** … ‘the ascending vagina, which was thin, oriented cranially, and included both a fused section attached to the urogenital cord and a free portion; and the descending vagina, which was thicker and ended at the vaginal sinus.

To make the text more informative, it may be rewritten as follows:…the ascending vagina, which was thin, oriented cranially, and included both a fused section attached to the urogenital cord and a free portion **oriented craneolaterally**, **followed by** the descending vagina, which was thicker and ended at the vaginal sinus.

Done.

**L337:** Figure 8 legend: ‘**Ub**’ for bladder, not ‘B’.

Done.

**L337:** Figure 8 legend: To complement the explanation of **image B**, the following or a similar statement may be added: “To visualize the lumen of the lateral vaginas, two rounded metal probes were inserted in a caudal-to-cranial direction, while a flattened probe was introduced in the same orientation into the centrally positioned birth canal.”

We have added a clarification in the legend of Figure 8 regarding the position of the rounded probes inserted into the lateral vaginas. However, we did not include the reference to a flattened probe in the central birth canal, as such an instrument was not used in this case.

**L346:** Figure 9 legend: Before explaining the labels, it may be added: “As an anatomical reference, the urethra runs ventrally” or a similar statement.

Done.

**L361-362:** ‘E. Detail of the regressing birth canal’. It may be completed adding: E. Detail of the regressing birth canal **displaying its mucosa at higher magnification.**

Done.

**L368:** …’it had a bifid glans and a groove extending from the bifurcation to the middle portion of the glans’. For further clarification, the following can be added: ’it had a bifid glans **oriented caudolaterally,** and’…

We have added a clarification to improve the description; however, we noted that the glans is oriented caudally rather than caudolaterally, and have adjusted the wording accordingly to reflect this anatomical detail accurately.

**L372-373:** ‘B. Detail of the clitoris. B, Base of the glans;… To avoid confusion with the image label, it could be designated as 'Ba' instead of 'B'. Additionally, to provide more information, the following may be added: Base of the glans ‘**oriented cranially’**…

Done.

**L376:** Instead of ‘Ugcf, folds of the urogenital canal’, it should read 'Ugcf, **mucosal** folds of the urogenital canal'

Done.

**L385:** … ‘In one juvenile and two adults, no teats could be identified, and smooth skin was found inside the skin fold (Figure 12C).’ The morphological description is shown in image **12D**, not in image 12C.

Done.

**L395-397:** ‘Regarding the number and arrangement of teats, significant variability was found, ranging from 0 to 11 teats, which could be button-shaped, papillary-shaped, or fully elongated.’ To complement the information provided, the following may be added: …**’depending on their physiological state**’: ‘Regarding the number and arrangement of teats, significant variability was found, ranging from 0 to 11 teats, which could be button-shaped, papillary-shaped, or fully elongated **depending on their physiological state’**…

Done.

**L406**: …‘while both subadults and juveniles had **no teats**.’… It should be clarified whether it is ‘**no teats’** or ‘**no inactive teats’.?????**

Done.

**L410:** …’In contrast, adult females had a considerably lower mean of 1.29 ± 2.26’. It should be added: In contrast, adult females had a considerably lower mean of **undelevolped teats** **(**1.29 ± 2.26**).**

Done.

**L412**: Table 3 is not included in the manuscript.

Thank you for pointing this out. The reference to Table 3 has been removed, as the table was ultimately not included in the manuscript due to its limited relevance to the main objectives of the study.

**L417:** … observing that the reproductive tract of *Didelphis marsupialis*… It may be appropriate to include the term 'female': …observing that the **female** reproductive tract of *Didelphis marsupialis…*

Done.

**L468:** It would be advisable to begin the sentence with: **'Regarding the oviduct’**, marsupials are reported to have…

Done.

**L495:** ‘In *D. marsupialis*, the perforated septum formed a vaginal sinus fold in the cranial portion’,… This vaginal sinus fold is placed dorsal and ventral, only dorsal or only ventral? Please, provide further specification or clarification.

Done.

**L530:** … ‘These findings suggest that the presence of epithelium in the birth canal may represent an intraspecific variation among marsupials.’ Only intraspecific? Or Intraspecific & interspecific?

Interspecific. It was corrected in the text.

Regarding the **References** (from **L610 onwards**):

Please review the hyphens used in the pagination, as some are short and others are long, which may indicate inconsistent usage. The same issue applies to the journal names: while most are written in full, some appear in abbreviated form (#5, 16).

Reference #2: Remove the parentheses from the pagination.

Reference #14: Incomplete. Is it a book chapter or a journal article?

Reference #24: Add parentheses around the year of publication.

Thank you for your careful review of the reference list. All references have been revised to ensure consistency and are now formatted according to the Vancouver style. This includes correcting hyphen usage in pagination, standardizing journal names, and addressing the specific issues noted in references #2, #14, and #24.

Reviewer #2:

The corrections in the manuscript were highlighted in blue.

Introduction:

1. I disagree that the term "unique" is the best way to define the developmental process of marsupials. I think "peculiar" sounds better.

The term was changed to ´peculiar´.

2. Marsupials are not kept in captivity for human consumption in Brazil. This is not a cultural practice in the country. Furthermore, I disagree that this is a justification for this study.

We appreciate the reviewer’s perspective. However, it is important to note that in certain regions of Brazil, particularly in Amazonas, opossums *are* used for human consumption. This cultural and subsistence practice, although not widespread across the entire country, does exist and has been documented. We believe that understanding the health and ecological aspects of species involved in such practices provides relevant and meaningful justification for this study.

3. At some points, the text sounds like automatically translated.

We appreciate the observation. We acknowledge that an automatic translation tool (ChatGPT) was used as an initial aid in translating the manuscript. However, the entire text was subsequently reviewed and manually edited to ensure accuracy and academic appropriateness.

Methodology

4. Since the methodology involves the use of cadavers collected by environmental agencies, it is important to mention how long after the animals' deaths they are still used for analysis. The practice of using dead animals for histological and anatomical analyses, even if "well-preserved," is controversial.

Of the 57 cadavers used in the study, 55 were stored in a freezer immediately after donation to preserve their condition. For histological analysis specifically, we used two freshly deceased specimens (less than 6 hours post-mortem), to ensure tissue integrity. We have added this information to the Methods section to clarify the time frame and handling procedures.

5. What bibliographic reference was used in the analysis and classification of the teats?

No references were found reporting variations or classifications of teats in marsupials, nor a detailed description of the pouch in opossums.

6. The author repeatedly cites "absence of ovarian structures" as a characteristic of immature ovaries. However, it is necessary to specify which structures are being referred to (probably those related to the ovarian cycle: ovarian follicles and corpus luteum). The ovaries have covering and supporting structures, for example, that exist independently of the ovarian cycle.

We understand that the term "ovarian structures" can be broad. However, in the context of this study, we use "ovarian structures" specifically to refer to those associated with the ovarian cycle—namely, follicles at various stages of development and corpora lutea. While we recognize that other anatomical components (e.g., connective tissue, surface epithelium) are also present in the ovary regardless of reproductive status, our use of the term is focused on structures indicative of functional or cyclical activity.

7. Only two specimens (adult and subadult) were selected for histological analyses of the complete reproductive tract. In addition to the very small n, other life stages and phases of the reproductive cycle were not considered. How is this decision justified? The article only shows the histology of some organs, but not the complete reproductive tract, as described in the methodology.

Obtaining fresh cadavers of wild animals suitable for histological analysis is challenging due to logistical and ethical constraints, which limits sample size. For this study, we prioritized the ovaries and birth canal, as these structures were central to our research questions. Although only two specimens were analyzed histologically, they represent key life stages (adult and subadult) that provide meaningful insights. We have clarified in the manuscript that the histological analysis focused on these specific organs rather than the complete reproductive tract, to better reflect the methodology used.

Results

8. Can the presence of young in the pouch be correlated with the time of year the animals were collected? Would these correlations apply to the number of young and the stage at which they were collected?

However, this study did not consider the time of year as a variable because the specimens were collected in Antioquia, Colombia, a region without marked seasonal variation. Therefore, correlating the presence of young in the pouch with the time of year is not applicable in this context. Additionally, the number and developmental stage of the young were recorded as observed but were not analyzed in relation to collection timing. We acknowledge that in regions with distinct seasons, such correlations might be relevant and encourage future research in such contexts.

9. Does the topic "reproductive tract morphology" refer only to adult animals? If so, this needs to be clearly stated, since we have several developmental variables relevant to the context of the study.

The study of the reproductive tract morphology was done in all the specimens (including the subadults and juveniles). We added the clarification in Table 2, that immature specimens correspond to juveniles and subadults.

10. Figure 3: Which segment of the fallopian tube is shown? From what life stage? In what reproductive phase?

The microphotograph in Figure 3 corresponds to the ampulla segment of the uterine (fallopian) tube, which was obtained from an adult specimen in the interestrus phase. We have clarified this information in the figure legend to ensure precise understanding.

11. Figure 2: Why weren't images for the "subadult" stage included?

The reproductive tract in the subadult stage was found to be very similar in morphology to that of the juvenile and adult stages. Therefore, to avoid redundancy, we chose to include only the juvenile and adult images in Figure 2. The figure was intended to highlight the main anatomical structures of the reproductive tract, which were clearly represented in those two stages.

12. Image A shows the infundibulum above and the ovary in the rest of the field. Highlighting the infundibulum in this image is unnecessary, especially since we don't have a corresponding image in the subadult animal (B). Comparing adult and subadult animals and showing the infundibulum in only one case creates confusion.
Removing the infundibulum from the field in image A would also make it unnecessary to label the "ovary" with the letter "O," as is currently done. The identification occurs in the cortical region of the ovary and does not encompass the entire organ fragment.

We appreciate the reviewer’s detailed comment. However, we believe it is important to retain the infundibulum in Figure 5A, as its presence helps to contextualize the spatial relationship between the ovary and surrounding structures. This may be of interest to some readers. We do not believe the image creates confusion, as the figure legend clearly states that panel A shows both the ovary and the infundibulum, while panel B shows only the ovary. Regarding the label "O" in panel A, it was repositioned to the center of the ovary, to improve clarity.

13. Figure 6: Which stage is shown?

Thank you for the observation. The aim of Figure 6 is solely to illustrate the morphological variations and to show all the structures of the female opossum reproductive tract. The reproductive stage of the specimens in correlation with the morphological variations is detailed in the main text and in Figure 7.

14. Figure 9/10: Which stage is shown? 15. Are only the adult and subadult stages being shown?

The aim of Figures 9/10 is solely to illustrate the morphological variations in the birth canal. The correlations between reproductive stage of the specimens and morphological variations are detailed in the main text and in Figure 7.

Discussion

16. Throughout the text, it becomes unclear which life phase or reproductive cycle the author is referring to. Since there are so many analyses and so much information, it is necessary to specify which phase or stage is being discussed.

We thank the reviewer for this important observation. We agree that specifying the life stage or reproductive phase is essential for interpreting the morphological variation described. Throughout the discussion, we have incorporated the physiological stage of the individuals into the analysis, where we correlate morphological features with reproductive status.

For example:

- In the **third paragraph**, we describe the presence of a long vaginal cul-de-sac in females during **estrus, interestrus, and even in one pregnant individual**.
- In the **fourth paragraph**, we discuss how females with **pouch young older than one week** showed only connective tissue in the urogenital cord, indicating regression of the birth canal.
- In the **ninth paragraph**, we explain that a **complete vaginal septum** is observed in **non-parous (immature) females**, while an **incomplete septum** is found in **adults at different reproductive phases**, suggesting changes associated with first parturition.
- In the **fourteenth paragraph**, we describe **epithelial remnants in the involuting birth canal** of a female with **pouch young older than three weeks**, which helped infer the mechanism of birth canal formation.
- Finally, in the **last paragraph**, we associate **elongated teats** with the presence of **pouch young**, and **papillary teats** with **diestrus** and **pregnancy**.

We hope this clarifies that reproductive phase and life stage were taken into account and integrated into the interpretation of anatomical findings.

17. Some sections of the discussion are very similar to the results and sounds repetitive. This is particularly true in sections where the findings are listed again before being discussed.

We thank the reviewer for this helpful observation. We agree that some sections of the Discussion initially repeated the Results too closely. To improve clarity and avoid redundancy, we have revised the text to focus more on interpretation and comparison with the literature.

- In the **third paragraph**, the sentence *“*unique and long vaginal *cul-de-sac* was present in females during estrus, interestrus, and even in one pregnant individual. In an individual with pouch young older than three weeks, a birth canal undergoing late involution was identified only in the cranial third of the urogenital cord with signs of cicatrization.*”* was replaced with: *“Our results regarding the morphology of the cul-de-sac and the presence of the birth canal may (…)”*.
- In the **fourth paragraph**, the sentence *“Additionally, we observed that females with pouch young older than one week had only connective tissue in the urogenital cord…”* was removed.
- In **paragraph 10**, the sentence *“*In *D. marsupialis*, the perforated septum formed a vaginal sinus fold in the cranial-dorsal portion, while a complete septum remained in the caudal portion, fully dividing the *cul-de-sacs*. When the birth canal was present, both the septum and the *cul-de-sacs* were absent.*”* was replaced by: *“In contrast, in D. marsupialis, the vaginal sinus displays multiple morphological presentations, with or without cul-de-sacs.”*
- Similarly, the **last paragraph** of the Discussion originally repeated findings related to teat morphology (*“*Elongated teats were commonly observed in individuals with pouch young, while papillary teats were found in individuals in diestrus and in the only pregnant individual.*”* was replaced by: *“Our results regarding the morphology of the teats (…)”.*

18. The discussion highlights the questionable methodology used with cadavers.

We appreciate the reviewer’s concern regarding the use of cadavers. We would like to clarify that all specimens were obtained through authorized environmental agencies and were handled following ethical guidelines and best practices for anatomical and histological research. Freshness and preservation were carefully considered: for histological analysis, only specimens received less than 6 hours postmortem were used to ensure tissue integrity, while others were frozen immediately upon receipt for anatomical description.

We acknowledge the limitations inherent in using cadaveric material, particularly for histology, but given the challenges of working with wild species, this approach remains one of the few ethically and logistically feasible options. We believe that the methods applied were appropriate for the study's objectives and yielded reliable and relevant anatomical and histological information.

19. The discussion compares the size of the isthmus and the ampulla of the uterine tube. However, no comparative histological analyses of the two segments were performed. The image of the uterine tube does not specify its specific segment or the animal's life/reproductive stage.

As noted, no comparative histological analyses between the ampulla and isthmus of the uterine tube were conducted, as the limited sample size did not allow for reliable morphoquantitative analysis. The comparison made in the Discussion is based on qualitative observations only.

To address this, we have clarified the limitation in the text and added the following sentence: *“Nevertheless, this characteristic must be confirmed by morphoquantitative analysis of this structure.”* Additionally, we have specified in the figure legend that the image corresponds to the ampulla of the uterine tube from an adult specimen in the interestrus phase.

20. The discussion of the epithelium of the birth canal could be further explored. The author comments that "there are differences between species," and this is notable. However, what functional and evolutionary aspects might be relevant in this context?

We agree that the functional and evolutionary aspects of the birth canal epithelium are relevant and merit further exploration. However, there is very limited information available in the literature regarding the birth canal in species where this structure is temporary, as is the case in many marsupials. This scarcity of comparative data constrains the depth of discussion on this topic.

21. Was histological analysis of the breasts considered?

Histological analysis of the mammary glands was not included, as it was not among the original objectives of the project and only a limited number of specimens were suitable for histology. However, we agree that this would be an interesting line of inquiry and suggest it as a topic for future studies.

22. Was analysis of the breasts considered when there were youngs at different stages of the pouch?

Histological analysis of the mammary glands was not included, as it was not among the original objectives of the project and only a limited number of specimens were suitable for histology. However, we agree that this would be an interesting line of inquiry and suggest it as a topic for future studies.
