# Supplementary material for: Reproductive tract and pouch anatomical variability across the reproductive phases in female common opossum (Didelphis marsupialis Linnaeus, 1758)
Source: PLoS One. 2025 Oct 9;20(10):e0334040. doi: 10.1371/journal.pone.0334040 (PMC12510574; doi:10.1371/journal.pone.0334040)
Supplement: Attachment — Submitted filename: PONE-D-25-30424-R1-comments.docx [file pone.0334040.s003.docx]

PONE-D-25-30424-R1

The authors have adequately addressed all the issues raised. Only two minor comments remain to be noted:

**-L240:** figure 4 legend: it should say ‘*corpora lutea’* instead of ‘*corpora luteum*’.

**-**Regarding **Figure 7 quality**: enhancing the contrast in this figure can be achieved as easily as using an image editing program such as *Photoshop*: by navigating to **Image** > **Adjustments** > **Curves** and modifying the **green channel** to intensify the pink tones with minimal impact on the other colors.

An example is provided:
